# An Anecdote of Waterloo

**Published:** 1882-05

**Authors:** 


### AN ANECDOTE OF WATERLOO.

Many are the anecdotes, many the daring deeds, many the acts of chivalry which have never found a place in books. Among the facts not made public, says an English paper, is the conduct of the Duke of Wellington before the battle of Waterloo. It is generally supposed that during the preceding night (June 17) Wellington supped and quietly slept in the village of Waterloo, some miles distant from the field of battle; the real fact is that, after the British army had bivouacked on the ground, the destined scene of the next day's fight, Wellington, unattended, rode to Blucher's headquarters and had an interview with him. His object was to secure the junction of the Prussians and the British as early as possible next day. Union was more important to the allies, while attack before this union should be effected was most important to Napoleon. Wellington and Blucher calculated that they could meet on the next day upon the field of Waterloo at twelve o'clock. After this interview, Wellington rode back to the village of Waterloo, where he obtained a short repose. Unfortunately, subsequent heavy rains rendered the road so nearly impassable that the junction was not accomplished until six in the evening of June 18. During those six hours of deep anxiety endured by the British chief, how often did he look at his watch! And, when at length the Prussian guns were heard, he involuntarily exclaimed, "There goes old Blucher at last." The two officers who alone knew of the midnight interview between Wellington and Blucher were Sir Augustus Fraser, R. A., and Captain (afterward General) William Bell, R. A., both of whom rode into the field with their chief, and both of whom have now departed this life.

### LITTLE DEEDS OF KINDNESS.

Each of a thousand acts of love costs very little of itself, and yet, when viewed together, who can estimate their value? The child whose good offices are always ready when wanted to run up stairs and down, to get chips, or rock the cradle, to run on an errand, and right back, all with a cheerful look and a pleasant temper, has a reward along with such good duties. If a little girl cannot take her grandfather on her lap as he takes her on his, she can get the slippers, or put away his book, or gently comb his thin locks; and whether she thinks of it or not, these little kindnesses that come from a loving heart are the sunbeams that lighten up a dark and woful world.

### A THOUSAND DOLLARS A MINUTE.

Mr. Eddy, the veteran patent solicitor of Boston, is a regular encyclopedia of incidents referring to inventors. He tells of a man named Hurd, who belonged in Stoneham, who realized $30,000, and gave to the world one of the most valuable inventions ever produced, all the result of only about half an hour's thought. His invention was the machine now everywhere used for extracting molasses from sugar. When the idea occurred to him he sketched it down and gave it to Mr. Eddy and authorized him to take out a patent. Returning home he forgot all about the matter and applied himself to other affairs. Subsequently a gentleman engaged in the sugar business saw the invention in Mr.

Eddy's office and at once appreciated its value. The solicitor was instructed to purchase the patent, which he supposed he could do for a moderate sum. The first offer of $1,000 was refused, and not until the figure of $30,000 was reached did Mr. Hurd surrender. The machine is used in all the sugar countries of the world. Mr. Robertson, who was the American Consul at Hague, and the Aspinwalls, of New York, made millions out of the invention.—*Boston Herald.*

